# Application repetition and electrode–tissue contact result in deeper lesions using a pulsed-field ablation circular variable loop catheter

**DOI:** 10.1093/europace/euae220

**Published:** 2024-08-16

**Authors:** Luigi Di Biase, Jacopo Marazzato, Tara Gomez, Eric Byun, Fengwei Zou, Vito Grupposo, Sanghamitra Mohanty, Vincenzo Mirco La Fazia, Giuseppe Ammirati, Aung Lin, Domingo Ynoa Garcia, Domenico Della Rocca, Amin Al Ahamad, Marco Schiavone, Alessio Gasperetti, Michael Freilich, Juan Cedeno Serna, Giovanni Forleo, Xu Liu, Dhanunjaya Lakkireddy, Claudio Tondo, Andrea Natale, Xiao-Dong Zhang

**Affiliations:** Department of Cardiology, Montefiore Medical Center, 111 E 210th Street, Bronx, NY 10467, USA; Department of Cardiology, Montefiore Medical Center, 111 E 210th Street, Bronx, NY 10467, USA; Electrophysiology and Cardiac Pacing Unit, Humanitas Mater Domini, Castellanza, Italy; Biosense Webster Inc., Irvine, CA, USA; Biosense Webster Inc., Irvine, CA, USA; Department of Cardiology, Montefiore Medical Center, 111 E 210th Street, Bronx, NY 10467, USA; Biosense Webster Inc., Irvine, CA, USA; Texas Cardiac Arrhythmia Institute, St David’s Medical Center, Austin, TX, USA; Texas Cardiac Arrhythmia Institute, St David’s Medical Center, Austin, TX, USA; Department of Cardiology, Montefiore Medical Center, 111 E 210th Street, Bronx, NY 10467, USA; Department of Cardiology, Montefiore Medical Center, 111 E 210th Street, Bronx, NY 10467, USA; Department of Cardiology, Montefiore Medical Center, 111 E 210th Street, Bronx, NY 10467, USA; Texas Cardiac Arrhythmia Institute, St David’s Medical Center, Austin, TX, USA; Texas Cardiac Arrhythmia Institute, St David’s Medical Center, Austin, TX, USA; Centro Cardiologico Monzino, Milan, Italy; Department of Cardiology, Johns Hopkins Hospital, Baltimore, MD, USA; Department of Cardiology, Montefiore Medical Center, 111 E 210th Street, Bronx, NY 10467, USA; Department of Cardiology, Montefiore Medical Center, 111 E 210th Street, Bronx, NY 10467, USA; Department of Cardiology, Luigi Sacco Hospital, Milan, Italy; Shanghai Chest Hospital, Shanghai Jiao Tong University, Shanghai, China; Kansas City Heart Rhythm Institute, Overland Park, KS, USA; Centro Cardiologico Monzino, Milan, Italy; Texas Cardiac Arrhythmia Institute, St David’s Medical Center, Austin, TX, USA; Department of Cardiology, Montefiore Medical Center, 111 E 210th Street, Bronx, NY 10467, USA

**Keywords:** Catheter ablation, Pulmonary vein isolation, Pulsed-field ablation, Irreversible electroporation, Pre-clinical model

## Abstract

**Aims:**

Pulsed-field ablation (PFA) is a novel, myocardial-selective, non-thermal ablation modality used to target cardiac arrhythmias. Although prompt electrogram (EGM) signal disappearance is observed immediately after PFA application in the pulmonary veins, whether this finding results in adequate transmural lesions is unknown. The aim of this study is to check whether application repetition and catheter–tissue contact impact lesion formation during PFA.

**Methods and results:**

A circular loop PFA catheter was used to deliver repeated energy applications with various levels of contact force. A benchtop vegetal potato model and a beating heart ventricular myocardial model were utilized to evaluate the impact of application repetition, contact force, and catheter repositioning on contiguity and lesion depth. Lesion development occurred over 18 h in the vegetal model and over 6 h in the porcine model. Lesion formation was found to be dependent on application repetition and contact. In porcine ventricles, single and multiple stacked applications led to a lesion depth of 3.5 ± 0.7 and 4.4 ± 1.3 mm, respectively (*P* = 0.002). Furthermore, the greater the catheter–tissue contact, the more contiguous and deeper the lesions in the vegetal model (1.0 ± 0.9 mm with no contact vs. 5.4 ± 1.4 mm with 30 g of force; *P* = 0.0001).

**Conclusion:**

Pulsed-field ablation delivered via a circular catheter showed that both repetition and catheter contact led independently to deeper lesion formation. These findings indicate that endpoints for effective PFA are related more to PFA biophysics than to mere EGM attenuation.

## Graphical Abstract

**Graphical Abstract:**
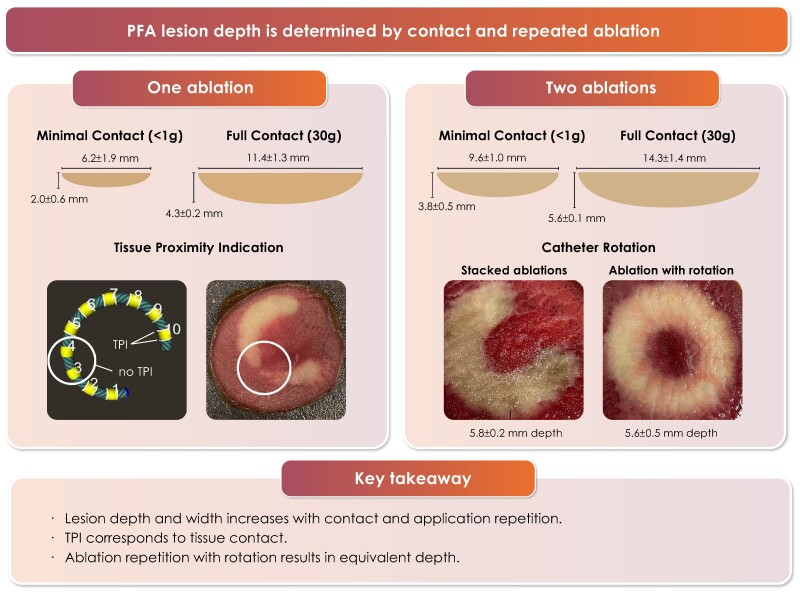

**Translational Perspective**

- A novel bipolar pulsed-field ablation (PFA) waveform has lesion extension into the inter-electrode spacing gaps, and increased contact force and application repetition each independently contribute to increased lesion depth.
- Evidence herein supports the generalizability of the biophysics contact force and application repetition have on lesion depth across PFA technologies.
- This represents the first evidence that a novel tissue contact indicator (tissue proximity indication) correlates with lesion contiguity with the PFA system characterized in this study.
- The vegetal and porcine model outcomes herein underscore recent clinical findings and workflow parameters reported elsewhere with the characterized PFA system.

## Introduction

Pulsed-field ablation (PFA) is a novel non-thermal ablative energy source relying on the mechanism of irreversible electroporation to cause selective myocardial cell death. Although PFA has proved to be safe and effective,^1–4^ a variety of PFA technologies have been developed over the years as proprietary recipes implementing different PFA catheters and biophysics. This progressively led to the lack of one universal model for studying PFA lesion creation and thereby creating the need to evaluate the dynamics of the lesion creation with each system. However, regardless of the system utilized, recent evidence showed that optimal PFA lesion formation may be dependent on both repeat PFA applications (application repetition) and catheter–tissue contact (contact force).

In fact, seemingly due to increased permeability across the cell membrane made by sequential application delivery,^3,4^ PFA application repetition seems associated with better lesion depth in pre-clinical models designed with focal^3^ and variable loop circular catheters (VLCCs).^5^ Likewise, in clinical trials, when the VLCC was implemented, treatment with fewer than 4 ablations per vein [48 total applications for a 4-vein pulmonary vein isolation (PVI)] per patient nearly doubled the rate of recurrence^6^ with similar outcomes obtained when a spherical globe catheter was utilized.^7^

In addition to PFA application repetition, adequate catheter–tissue contact seems to improve lesion depth in pre-clinical studies where both focal^8^ and basket catheters^9^ are implemented during PFA. However, current evidence for VLCCs is missing in this regard.

Moreover, when VLCCs are used for PVI, it is common practice to observe the prompt disappearance of electrogram (EGM) signals immediately after PFA delivery. Whether this observation is associated with adequate lesion depth to achieve tissue transmurality and better long-term outcomes after ablation is still unknown.

Therefore, in this study, a fully integrated PFA system comprising a proprietary PFA generator, a novel VLCC, and a compatible mapping system was used to understand the dynamics of lesion creation with respect to the number of sequential applications delivered, contact, and force. We believe that these results may provide insights into the most effective clinical endpoints to be considered during PFA (i.e. application repetition and optimized catheter–tissue contact vs. the mere EGM signal attenuation).

Finally, given that the use of the thigh muscle model poses challenges to a cardiac PFA system due to tissue specificity and other energy-specific differences, both a potato vegetal model and a ventricular myocardial model were developed within this study in accordance with prior literature.^10^

## Methods

### Study design

Both a benchtop vegetal potato model and a beating heart ventricular myocardial model were utilized to evaluate the biophysics of PFA lesion formation. Both models were utilized to assess the impact of total PFA dosage (repetition of PFA applications) on the depth of lesions formed. Contact force and the impact of repositioning were assessed using the vegetal model only, as the degree of contact and catheter placement could be controlled on the benchtop with greater precision than in the beating heart. A comparison of the two study methods is presented in *Table 1*.

**Table 1:** A summary of porcine ventricle lesion experiments

	Vegetal model	Animal model
Total *n* (lesions)	*n* = 144 lesions	*n* = 53 lesions
Target tissue	Russet potato	Porcine ventricle myocardium
Study type	*In vitro* (benchtop)	*In vivo* (beating heart)
Test parameters (independent variables)	PFA dosage, contact force, repositioning vs. stacking	PFA dosage
PFA doses tested (ablations per lesion)	1×, 2×, 3×, 6× ablations (each defined as three consecutive applications); as well as incomplete ablations of 1× and 2× applications	1× and 2× ablations
Contact forces tested	No contact, <1, 15, 30 g	Consistent contact (TPI, EGM, and ICE validated)
Repositioning vs. stacking tested?	Yes	No
Study endpoint (dependent variable)	Max lesion depth in cross-sectional slices of potato	Max lesion depth in cross-sectional slices of the ventricle
Lesion stain	TTC	TTC

EGM, electrogram; ICE, intracardiac echocardiography; PFA, pulsed-field ablation; TPI, tissue proximity indication; TTC, triphenyl tetrazolium chloride.

### Ablation parameters

Pulsed-field ablation was performed with a fully integrated PFA system including the VLCC mapping and ablation catheter (VARIPULSE), a PFA generator (TRUPULSE), and a CARTO 3 mapping system (Biosense Webster, Irvine, CA, USA). An irrigation pump (nGEN) and standard tubing, pacing system, and requisite cables were used.

Each PFA application includes trains of biphasic bipolar pulses delivering 1800 V within 250 ms. A 10 s pause was maintained between each delivered application per clinical findings. A constant irrigation flow rate of 4 mL/min was maintained during the procedure.

### Vegetal potato model

To prepare the vegetal model, russet potatoes were sliced horizontally to achieve round core slices of 10 mm thickness and kept in distilled water until use to prevent oxidation. For data collection, these potato slices were secured in a warm saline bath (conductivity of 6.5 ± 0.5 mS/cm). The VLCC was secured to a laboratory stand with clamps and a swivelling catheter arm with counterweights to adjust the force with which the catheter tip contacted the sample while the arm was level. A laboratory scale–level with the height of the potato slice was used to confirm the contact force with which the catheter was touching the potato substrate.

Pulsed-field ablation was performed with various numbers of stacked energy deliveries: 1×, 2×, 3×, and 6× consecutive ablations (each defined as 3 applications) as well as incomplete ablations of only 1 and 2 applications. These ablations were performed under various contact forces (<1, 15, and 30 g, and complete non-contact with ∼2 mm distance between catheter electrodes and potato). Also, 2× consecutive ablations were delivered with and without a repositioning of the catheter after the first ablation, which mimics a standard clinical ablation workflow. Contact force and repositioning were tested only in the vegetal model to take advantage of the controlled bench environment. As the VLCC does not include a built-in force sensor, *in vivo* force calculation is not possible.

After ablation, the potato slices were incubated in a 1% w/v solution of triphenyl tetrazolium chloride (TTC) for 18 ± 0.5 h. Each potato core was then sliced into ∼2 mm lateral slices to expose the lesion depth at various points within the lesion. High-resolution photographs were taken of the lateral slices with a calibrated stainless-steel ruler included for scale. The lesion images were then assessed for depth using Fiji ImageJ software using electronic callipers, with the maximal depth of each lesion recorded. No lesions were transmural (exceeding the 10 mm thickness of the potato slice) in this data set.

### Animal model

Eight Yorkshire swine (64 ± 7.7 kg) were studied under general anaesthesia with mechanical ventilation. The care and use of animals was conducted in accordance with the regulations of the US Department of Agriculture Animal Welfare Act. The research protocol was approved by the Institutional Animal Care and Use Committee and conformed to the Position of the American Heart Association on Research Animal Use.

All procedures were performed under anticoagulation with heparin with an activated clotting time range of 300–400 s. To assess the EGM behaviour during PFA, mapping of the left atrium was carried out in all swine and the right superior and inferior pulmonary veins (RSPV and RIPV) were ablated in all animals that were euthanized humanely 7–10 days post-treatment. Lesion depth was evaluated pre-clinically in the pulmonary veins, but the thin myocardial tissue at these sites constrains the maximum observable lesion depth. Therefore, to better assess lesion depth and contiguity, ventricular ablations were performed as described.^4^ In summary, a diagnostic catheter (PENTARAY) was used to map both the right ventricle and the left ventricle of the porcine subjects. The VLCC loop was placed in contact with the endocardial surface of the ventricles with tissue proximity indication (TPI), EGM signal amplitudes, and intracardiac echocardiography (ICE) used to validate the presence of tissue contact for ablation. Between each lesion, a distance of ∼10 mm was measured wherever possible and six active electrodes were used for all applications to reduce the incidence of overlap between distinct lesions and optimize the space within the same ventricle. Per chamber, applications were delivered as a single ablation [defined identically to the vegetal model as 3 applications per site (3×), or as a sequence of two consecutive ablations, meaning 6 applications per site (6×)]. Partial ablations could also be delivered by delivering non-multiples of 3, i.e. 1 application is 0.33 ablations. Each lesion was tagged on the CARTO map so that it could be correlated to its settings during histopathology. After ventricle ablation, all study animals were allowed to survive for no less than 6 h prior to humane euthanasia.

### Porcine gross pathology

Prior to euthanasia, TTC was administered. The ventricles were harvested and fixed in formalin for ≥7 days. Each porcine heart was opened longitudinally along the long axis of the ventricles to view the endocardial surface. Each ventricular lesion was identified based on the CARTO map and cut into lateral slices to expose the cross-sectional depth, with fully transmural lesions excluded. The lesions were characterized as greyish white tissue. Using a microscope, the lesions were photographed, and the depth of each lesion was measured using electronic callipers.

### Porcine histopathology assessments

Formalin fixed–treated tissue samples were photographed and trimmed. All trimmed ablation sites were embedded in paraffin and sectioned at 4 µm thickness. The samples were stained with haematoxylin, eosin stain, and Masson’s Trichrome stain before microscopic scoring by a licensed pathologist. The data undelying this article will be shared on reasonable request to the corresponding author.

## Results

### Single applications of pulsed-field ablation can silence electrogram activity in a pulmonary vein but make shallow lesions

**Figure 1:**
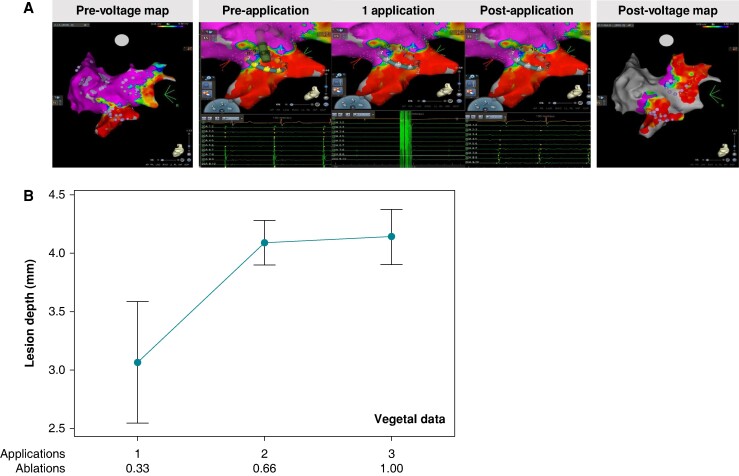
Single applications of pulsed-field ablation can silence electrogram activity in a pulmonary vein, but make shallow lesions. (*A*) A CARTO display of a porcine right inferior pulmonary vein. Left: A pre-ablation voltage map are shown. Middle: A single application of pulsed-field ablation is delivered, and noise depicts the moment of energy delivery. Right: After just 1 application of energy at the right inferior pulmonary vein, electrogram signal attenuation of the vein isolation is noted (1 application, 0.33 ablations). A post-voltage map is shown. As depicted by the number of purple ablation tags, the porcine subject was ultimately treated with two applications at the right inferior pulmonary vein and one application at the right superior pulmonary vein. In both cases, an increase in low-voltage area is observed relative to the pre-voltage map. Images courtesy of © Biosense Webster, Inc. All rights reserved. (*B*) A scatter plot of lesion depth in a vegetal model when 1–3 applications (0.33–1 ablation) are delivered. As observed in the panel, the greater the number of applications, the deeper the lesion.

Single PFA applications led to prompt EGM disappearance in all animals at the RIPV and RSPV. Post-ablation voltage mapping of the ablated veins also showed low-voltage areas of scar tissue (*Figure 1A*). Histology performed on the representative subject in *Figure 1A* showed hallmarks of PFA, as the lesions were non-necrotic with limited inflammation and mineralization (*Table 2*). However, in the vegetal model, single PFA applications were associated with significantly shallower lesions when compared with repeat applications, from 1.8 ± 1.3 mm to a maximal lesion depth of >4 mm when single and two or three PFA applications were delivered, respectively (*Figure 1B*).

**Table 2:** A summary of porcine atrial and ventricle lesion histopathological evaluation

Evaluation	Atrial tissue^a^		Ventricular tissue^b^		
	RIPV (2 applications)	RSPV (1 application)	Tissue sample #1	Tissue sample #2	Tissue sample #3
Myocardial ablation contiguity % on the endocardial surface	N/A	N/A	67.91	75.22	92.67
Thermal necrosis	NP	NP	NP	NP	NP
Mural inflammation	Moderate	Mild	Moderate peripheral inflammatory infiltration	Mild peripheral inflammatory infiltration	Moderate peripheral inflammatory infiltration
Mineralization	Mild	Minimal	NP	NP	NP
Endocardial fibrosis	Moderate	Mild	NP	NP	NP

NP, not present; N/A, not assessed; RIPV, right inferior pulmonary vein; RSPV, right superior pulmonary vein.

^a^A histology of a representative porcine subject shown in *Figure 1A*.

**Figure 2:**
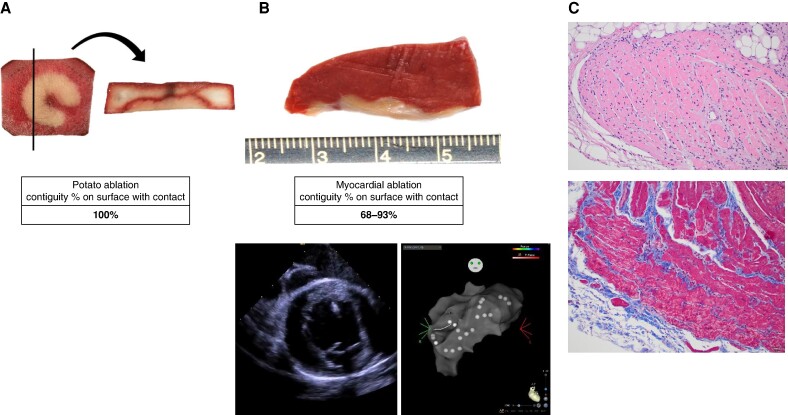
Lesion contiguity is achieved between the inter-electrode spaces. (*A*) A vegetal model sample of one ablation. In the vegetal model, the cross section displays a fully contiguous lesion profile, represented by the convex area below the line. Natural discolouration of the potato has resulted in a dark area in the middle of the potato slice above the lesion region. (*B*, top) A porcine model sample of one ablation. (*B*, bottom) Representative intracardiac echocardiography and CARTO images demonstrate the study methodology in the ventricle; contact was confirmed with intracardiac echocardiography before energy application. Images courtesy of © Biosense Webster, Inc. All rights reserved. (*C*, top) A high-magnification photomicrograph of ventricle ablation from *B*. The majority of the myocardial cells has smooth, eosinophilic cytoplasm with wavy hyper-eosinophilic bands (their cytoplasm has lost its cross striations), and their nuclei exhibit pyknosis (nuclear condensation). Haematoxylin and eosin staining; the scale bar equals 50 µm. (*C*, bottom) A high-magnification photomicrograph of ventricle ablation from *B*. Myocardial cells in the lower portion of the image have fragmented and finely vacuolated cytoplasm (their cytoplasm has lost its cross striations). Haematoxylin and eosin staining; the scale bar equals 20 µm.

^b^A histology of a representative porcine subject in *Figure 2B* (Tissue sample #1) and other subjects (Samples #2 and #3).

### Lesion contiguity is achieved between the inter-electrode spaces

The VLCC has 4 mm spaces between each of its 10 electrodes. Both vegetal and porcine lesions were assessed to query if lesions form within these inter-electrode spaces. In the porcine heart, CARTO visualization and ICE were utilized to ensure contact prior to energy delivery (*Figure 2B*). In the vegetal model, 100% lesion contiguity was observed on the potato surface when 30 g contact was made (*Figure 2A*). In the porcine ventricular model, lesion formation in between electrodes was also noted visually and with microscopic histological analysis of tissue samples. Ventricular tissue samples treated with one or two ablations ranged in endocardial surface contiguity, from 68 to 93%, indicating that lesions formed in the spaces between ablation electrodes (*Figure 2B*). Histologically, the ablated areas in the myocardium were characterized by loss of cytoplasmic cross striations, cytoplasmic hypereosinophilia, cytoplasmic granularity, and karyopyknosis (characteristic histological hallmarks of acute PFA lesion). There was some mild-to-moderate inflammation even when high contiguity was achieved that could be found in these tissues 6 h post-ablation, although there were no areas of necrosis, tissue mineralization, or endocardial thrombosis (*Figure 2C* and *Table 2*).

### Application repetition results in increased lesion depth in the vegetal and porcine model

**Figure 3:**
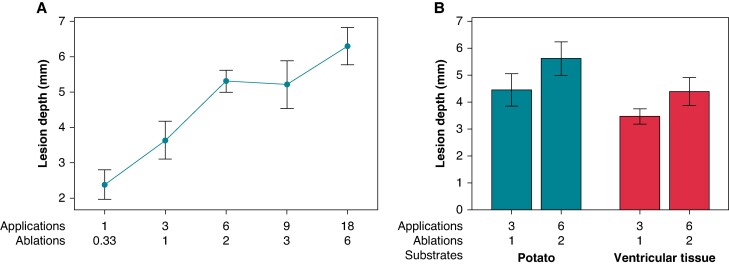
Application repetition results in increased lesion depth in a vegetal (*A*) model and porcine (*B*) model. (*B*) A bar graph comparing lesion depths in both vegetal and porcine ventricle tissue when either three or six applications are stacked.

A progressive increase in lesion depth was observed with application repetition using the VLCC system. In the vegetal model using all 10 electrodes, lesion depth varied between 1.8 ± 1.3 mm with just 1 application of energy, up to 5.1 ± 2.3 mm with up to 18 applications of energy delivered (*Figure 3A*). In animal subjects with a 6 h survival, lesion depth also showed a dependency on the number of delivered applications when using six electrodes. When one ablation of three stacked applications was delivered, the mean lesion depth was 3.5 ± 0.7 mm. The mean lesion depth when two ablations (six applications) were stacked was 4.4 ± 1.3 mm (*P* = 0.002; *Figure 3B*). The trend of repetition dependency on depth was consistent, and lesions were similar between the vegetal and ventricular model (*Figure 3B*). A plateau effect was observed in the vegetal model beyond two consecutive ablations. There was no significant increase in lesion depth observed when three ablations were stacked (4.3 ± 0.4 mm mean depth) compared with two (4.6 ± 0.3 mm mean depth). Tripling the number of stacked ablations from two to six (for a total of 18 applications) resulted in <1 mm of additional lesion depth on average (from 4.6 ± 0.3 mm with two ablations to 5.1 ± 0.5 mm with six; *Figure 3A*).

### Contact force results in increased lesion depth

**Figure 4:**
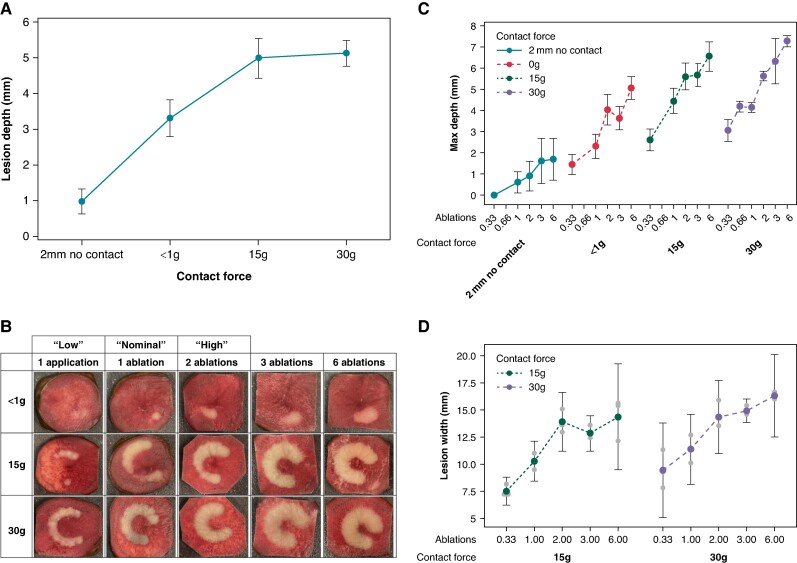
Contact force results in increased lesion depth. (*A*) An interval plot of lesion depth vs. catheter contact in a vegetal model (*P* < 0.001). (*B*) Images of potato lesions corresponding to one application up to six ablations with 0, 15, and 30 g of contact force. The terms ‘low’, ‘nominal’, and ‘high’ refer to the number of energy applications delivered before moving or rotating the catheter. Low dose entails one application, nominal dose entails three applications/one ablation, and high dose entails six applications/two ablations without moving the catheter. (*C*) An interval plot of lesion depth vs. contact force (*P* < 0.001) and ablation dosage (*P* < 0.001). (*D*) An interval plot of lesion width vs. contact force (*P =* 0.228) and ablation dosage (*P* < 0.001).

Contact force correlated to lesion depth in testing with the VLCC system. When a single ablation (three applications) is delivered with no contact, and with the catheter 2 mm above the surface of the vegetal model, a lesion only 0.6 ± 0.5 mm deep is formed. When the VLCC was placed in contact with the vegetal surface but with no weight added to the lever arm to apply force, a lesion of 2.3 ± 0.6 mm was observed. When weights were added to push the VLCC with either 15 or 30 g of force, lesion depth of 1 ablation reached 4.3 ± 0.4 mm, although there was some plateau of the effect at those forces (*Figure 4A*). Assessing both the impacts of contact force and application repetition together, better lesion contiguity and wider lesions are noted visually on the surface of the vegetal model (*Figure 4B*). When plotted for lesion depth, both the trends of application repetition and contact force are evident, with the most shallow lesions being made when no contact and fewer applications are applied (*Figure 4C* and *Table 3*). The lesion width response is similar to the lesion depth response, with a strong correlation between application repetition and lesion width (*P* < 0.001). The width of lesions made with 15 g consistently trended slightly smaller than the width of lesions made with 30 g, although the difference did not reach statistical significance (*P* = 0.228) (*Figure 4D*).

**Table 3:** A summary of lesion depth under various ablation doses and contact force conditions

Dose	Contact force			
	No contact	0 g	15 g	30 g
1 application (low)	0.0 ± 0.0	1.5 ± 0.4	2.6 ± 0.5	3.1 ± 0.5
2 applications	N/A	N/A	N/A	4.2 ± 0.4
1 ablation (nominal)^a^	0.6 ± 0.5	2.3 ± 0.6	4.4 ± 0.6	4.1 ± 0.2
2 ablations (high)	0.9 ± 0.7	4.0 ± 0.7	5.6 ± 0.6	5.6 ± 0.4
3 ablations	1.6 ± 1.0	3.6 ± 0.5	5.7 ± 0.5	6.3 ± 1.0
6 ablations	1.7 ± 0.9	5.1 ± 0.5	6.5 ± 0.7	7.3 ± 0.9

Data presented as mean ± standard deviation in mm.

^a^One ablation is equal to three applications.

### Tissue proximity indication corresponds to contact and lesion contiguity

**Figure 5:**
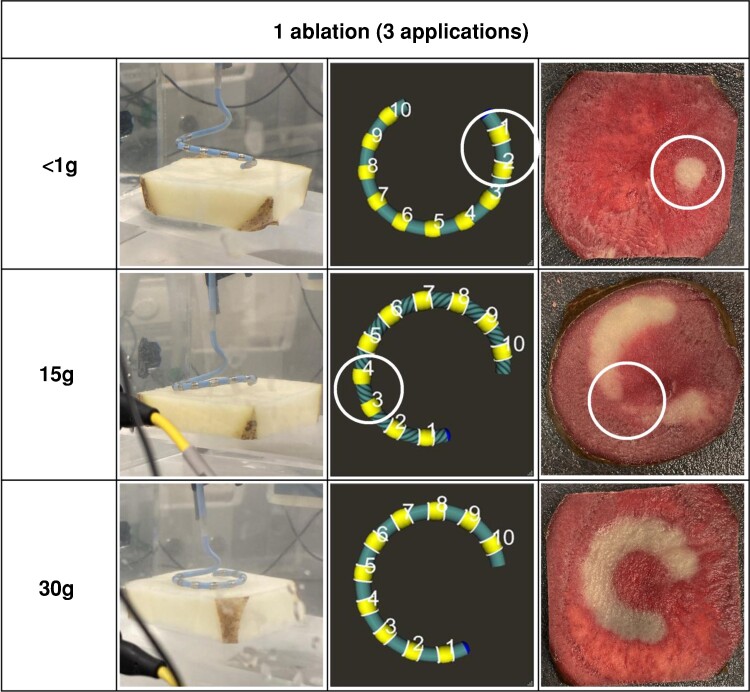
Tissue proximity indication corresponds to contact and lesion contiguity. Images of potato lesions corresponding to one ablation with 0, 15, and 30 g of contact force. Electrodes with white border rings indicate positive tissue proximity indication. Circled are corresponding areas on the tissue proximity indication and the lesion itself. In areas where there was negative tissue proximity indication, a lack of lesion contiguity is noted. Images courtesy of © Biosense Webster, Inc. All rights reserved.

The VLCC in conjunction with the CARTO mapping system has a TPI feature. When the VLCC was pressed with increasing force against the surface of the vegetal model, lesions could be seen at the locations corresponding to positive TPI. Gaps in positive TPI resulted in gaps in the created lesions, and positive TPI on all electrodes resulted in a fully contiguous lesion (*Figure 5*). In the porcine myocardial model, contact on all six active electrodes was validated using TPI, but the three-dimensional morphology of the porcine ventricle can result in non-contiguous lesions unrelated to contact force, so no contiguity assessment was performed.

### Variable loop circular catheter repositioning produces similar lesion depths as application stacking

**Figure 6:**
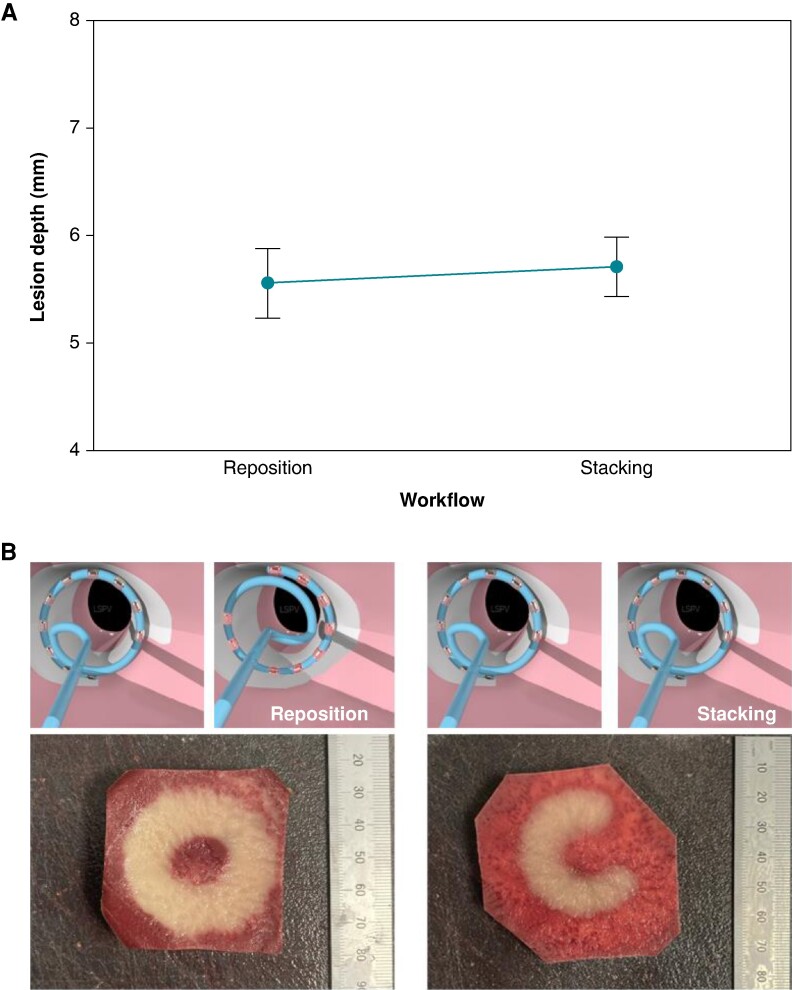
Variable loop circular catheter repositioning produces similar lesion depths as application stacking. (*A*) An interval plot of lesion depth vs. catheter workflow (*P* = 0.444). (*B*, left) An illustrative depiction of catheter workflows; the variable loop circular catheter is repositioned to obtain a rotation of the loop. (*B*, right) The variable loop circular catheter is held in place while delivering two ablations. A comparison of vegetal model samples with two ablations with repositioning (right) and two stacked ablations (left). Notice that the gap between Electrodes 1 and 10 where no lesion forms has been filled by loop rotation in the repositioning workflow. Images courtesy of © Biosense Webster, Inc. All rights reserved.

As mentioned previously, application stacking creates deeper lesions. However, the workflow for the VLCC when used in clinical practice^6^ involves applying two ablations: one at an ostial and another at an antral position with a repositioning step to rotate the loop of the VLCC. On the vegetal model, lesions made from two ablations with either a strategy of repositioning or stacking achieved similar depths of 5.6 ± 0.5 or 5.7 ± 0.4 mm, respectively (*Figure 6A* and  *B*).

### A repositioning to achieve a 120–150° rotational angle closes the 1–10 electrode gap

**Figure 7:**
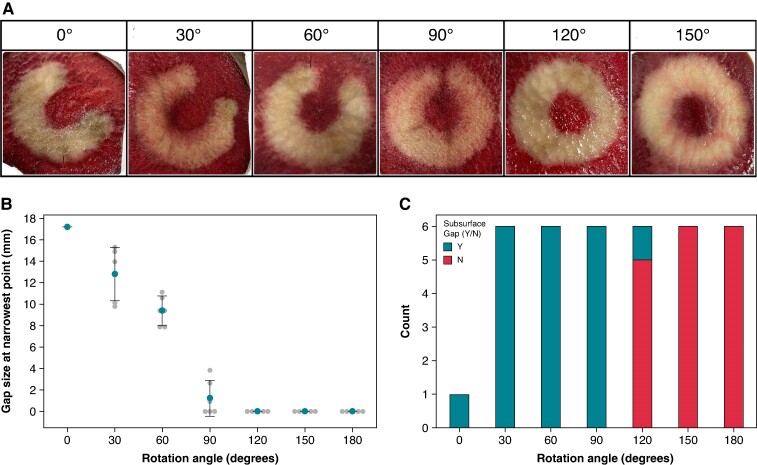
Variable loop circular catheter repositioning resulting in an angular rotation of 120° or more closes the 1–10 electrode gap. Images are of two stacked ablations on a potato model. (*A*) The degree measures indicate the angle to which the tip of the variable loop circular catheter is rotated (0–180°) around the centre point of the loop from the position of the first ablation to the position of the second stacked ablation. (*B*) A plot of the width of the gap (where present) on the surface of the potato core slice after two stacked ablations with the indicated rotation angle in between energy deliveries. (*C*) A comparison of the occurrence of subsurface gaps in the vegetal model after the two stacked ablations with the indicated rotation angle.

When the loop of the VLCC is uncontracted, there exists a physical gap between Electrodes 1 and 10, where no bipolar energy is delivered. Given that the workflow involves one ablation followed by a repositioning of the catheter before a second ablation, the necessary rotational angle required to entirely encompass the gap between electrodes was queried. When delivering ablations with a fully uncontracted loop, a rotational angle of at least 120° resulted in a fully contiguous, circumferential lesion and reduced the size of the unablated area left between Electrodes 1–10 (*P* < 0.001; *Figure* *7A* and *B*). However, cross sections of the vegetal core slices indicated that subsurface gaps—regions of shallower depth and thus less efficacious lesions—could remain unless a rotation of at least 150° was performed (*Figure 7C*).

## Discussion

Given that a single application of PFA energy can silence a pulmonary vein, it creates an understanding that the clinically relevant endpoints of a PVI procedure with PFA should be distinct from the endpoints used with traditional radiofrequency ablation.^11^ Electrogram signal attenuation may appear silenced in a pulmonary vein (PV) that does not have enough energy to adequately create the depth of lesion required for long-term success.^12^ A prior publication also found that a single application of another PFA waveform resulted in complete elimination of all high-frequency potentials.^3,4^ Hence, there is an urgent need to consider clinical endpoints for PVI apart from EGM attenuation. As such, anatomical placement of lesion sets with an adequate evidence-defined density and catheter–tissue contact should be the endpoint for PVI treatment.

Recent studies have demonstrated the correlation of contact force and application repetition to lesion depth.^9,13^ Here, we also demonstrated that bipolar PFA had lesion extension into the inter-electrode spacing gaps and that the dynamics of this lesion formation was firmly reliant upon contact force and application repetition. Moreover, both the vegetal and porcine models were used to demonstrate the biophysics of these effects. These models each posed multiple advantages. In particular, ventricular tissue is thicker than atrial tissue, allowing more precise measurements of lesions that are deeper than typical atrial tissue, and the potato model has a history of use for understanding the bioelectrical aspects of PFA.^3,10^

The VLCC has a defined workflow that treats one ablation as a delivery of three applications and requires two ablations with repositioning at both ostial and antral levels of the PV. Prior VLCC dose–response data helped elucidate how three applications created optimal acute and chronic outcomes in a pre-clinical model when compared with dosages using more or fewer applications.^5^ Here, we show that beneficial outcome is likely the achievement of maximal lesion depth by the third application of energy. Again, there appears to be a generalizability across multiple PFA waveforms to these biophysical outcomes. However, every technology should be individually evaluated based on the unique waveform and catheter design to define the exact number of applications required to achieve transmurality and the thresholds where no further energy deliveries will provide clinical benefit.

This work also shows the correlation between the vegetal and the porcine models. As the number of animals used in ablation technology research has been progressively reduced for bioethical issues, the similarity of the lesion depths achieved in both of these models indicates such can be done. Of note, the vegetal model data were prepared using 10 electrodes, while the ventricular lesion depths were made with 6 electrodes. Although the data are quite similar, the variation in the number of active electrodes could itself help explain the subtle differences between the maximum lesion depths achieved. On average, slightly deeper lesions were achieved using the vegetal model. In addition to the use of all 10 electrodes in the vegetal model, this effect may also be due to the perfect consistency of contact achievable on an external bench model vs. any biological constraints to perfect contact in the porcine model.

The limitations of our study are unknown correlations between ventricular and atrial results, correlation to humans, and the high trabeculation of ventricular tissue resulting in a less homogeneous surface for uniform contact than would be the case when ablating atrial tissue. Additionally, while the trend in lesion depth as a result of application repetition is similar between the ventricular and the vegetal models, it is unknown whether all factors can be extrapolated, as contact force cannot be measured in the beating heart with the VLCC. Finally, the impact of eccentric positioning and configurations of the VLCC loop at the pulmonary vein should be evaluated in the future for its impact on efficacy.

## Conclusions

This study indicates that with the VLCC, contact, force, and application repetition are important for lesion depth. Furthermore, the evidence for the VLCC workflow is supported by the data herein, where repositioning results in lesions just as deep as stacking, and three applications represent an optimal ablation dose.

## Data Availability

The data underlying this article will be shared on reasonable request to the corresponding author.
